# Microglial Expression of Serotonin Receptors Reveals Parallel Regulation of 5-HT2b and BDNF in the Rat Hippocampus

**DOI:** 10.3390/cells15010066

**Published:** 2025-12-30

**Authors:** Andrei Turkin, Maria Sidorova, Ekaterina Kurilova, Natalia Alenina, Oksana Tuchina, Friederike Klempin

**Affiliations:** 1 Institute of Medicine and Life Sciences (MEDBIO), Immanuel Kant Baltic Federal University, Kaliningrad 236035, Russia; turkinandy@gmail.com (A.T.); masidorova1@kantiana.ru (M.S.); ekurlova1@kantiana.ru (E.K.); 2 Max Delbrück Center for Molecular Medicine in the Helmholtz Association, 13125 Berlin, Germany; alenina@mdc-berlin.de; 3 Department of Psychiatry and Psychotherapy, Charité University Medicine Berlin, 10117 Berlin, Germany; 4 LSU Health Sciences Center, Department of Neurology, New Orleans, LA 70112, USA

**Keywords:** serotonin, 5-HT2b, Tph2, microglia, BDNF, inflammation

## Abstract

**Highlights:**

**What are the main findings?**

- Transient, region-specific microglial 5-HTR expression;
- Upregulated microglial 5-HT2b in *Tph2*^−/−^ rats parallels BDNF levels.

**What are the implications of the main findings?**

- Temporal differences in 5-HTR signaling may drive adaptive responses to serotonin deficiency, with BDNF compensating at the later stage;
- Interplay between microglial serotonin and BDNF highlights potential targets for interventions in neuroinflammation.

**Abstract:**

Growing evidence suggests that psychiatric disorders are characterized by a prolonged inflammatory state, which may influence the efficacy of compounds targeting serotonin. Serotonin is a key signaling molecule in neuroplasticity of the adult hippocampus and involved in antidepressant action. Recent in vitro studies indicate the neurotransmitter may also facilitate the response to inflammation and potentially modulate microglial function towards neuroprotection. Using *Tph2*^−/−^ rats depleted of brain serotonin, we examined microglial expression of various serotonin receptors (5-HTRs) in vivo in both the hippocampus and prefrontal cortex and assessed mRNA levels of cytokines and brain-derived neurotrophic factor (BDNF). We observed age-dependent and region-specific gene expression of 5-HTRs on sorted microglia, paralleling changes in BDNF signaling, especially with 5-HT2b. Notably, both 5-HT2b and BDNF expression in the hippocampus was significantly upregulated in the absence of brain serotonin. Our data indicate distinct roles of 5-HTR subtypes in early network formation (5-HT1b, 5-HT5b) and in the response to endogenous changes (5-HT2b, 5-HT5a). Understanding serotonin–microglia interplay could offer therapeutic insights into the maintenance of mood via brain–immune cell interactions.

## 1. Introduction

Microglia, the brain’s endogenous immune cells, contribute to neuroplasticity of the hippocampus under physiological conditions and orchestrate the acute inflammatory response to pathogenic stimuli. Specifically, microglia control the neuronal cell pool [1] and participate in learning-dependent synaptic plasticity [2] by engaging in local signaling pathways, such as the brain-derived neurotrophic factor (BDNF), which is important in memory formation [3]. Furthermore, microglia adopt a neuroprotective, anti-inflammatory phenotype following physical exercise [4,5]. Upon stimulation, microglia express cell surface molecules, release cytokines, and initiate phagocytic activity. Among cytokines, interleukin (IL)-1β and tumor necrosis factor-a (TNF-a) have pro-inflammatory functions [6,7], while IL-10 and BDNF primarily exert anti-inflammatory and neuroprotective effects [8]. Chronic activation of microglia can lead to prolonged inflammation, which is increasingly implicated in the progression of neurodegenerative diseases and psychiatric disorders [9].

In major depression, pharmacotherapy often involves serotonin-targeting medications, which acutely enhance serotonin transmission. However, clinical improvement typically occurs only with chronic treatment, and the efficacy of these drugs varies among patients, potentially due to an underlying inflammatory state. Serotonin exerts its effects through a variety of serotonin receptor (5-HTR) subtypes located on neurons and interneurons, which modulate the response to efferent activity. Target areas in the dentate gyrus of the hippocampus predominantly express 5-HT1a, 5-HT2a, 5-HT2c, and 5-HT5a [10,11,12]. Recent in vitro studies and research on acute brain slices show that microglia also express receptors for classical neurotransmitters, suggesting their involvement in neuronal network excitability [13,14]. Notably, microglial cells strongly express 5-HT2b, indicating that serotonin signaling may play a role in regulating microglial activity and function [15].

Here, we utilized rats lacking brain serotonin due to the genetic deletion of tryptophan hydroxylase 2 (TPH2). *Tph2*^−/−^ rats were examined at postnatal days (P)9, P21, and P56 to investigate serotonin-dependent microglial function both at a young age and in adulthood in vivo, thereby focusing on neuroplasticity and inflammation. We analyzed the mRNA expression profiles of 5-HTRs in fluorescence-activated sorted (FACS) microglia from the hippocampus and prefrontal cortex while also evaluating cytokine levels and BDNF signaling. We hypothesize that microglia, through their expression of 5-HT2b, may play a dual role in modulating neuroimmune plasticity.

## 2. Materials and Methods

### 2.1. Animals

Animal experiments were approved by the local animal welfare and ethical review body (Landesamt für Gesundheit und Soziales, LAGeSo, Berlin, no. G0047/14, 15 July 2014) and conducted in compliance with requirements set out in the European Communities Council Directive 2010/63/UE. Experiments and the number of animals used in this study were also approved by the Independent Ethical Committee of the Clinical Research Center at Immanuel Kant Baltic Federal University (IKBFU), Kaliningrad, protocol 27/2021, 2 November 2021.

*Tph2*^−/−^ transgenic rats were generated on the Dark Agouti background using zinc finger nuclease technology, as detailed previously [16]. To investigate microglial expression of receptors and signaling factors, a total *n* = 42 male and female *Tph2*^−/−^ rats and heterozygous littermates (CTR) were utilized at ages P9 (n = 15: male 4 CTR, 3 *Tph2*^−/−^, female 4 CTR, 4 *Tph2*^−/−^), P21 (n = 15: male 4 CTR, 3 *Tph2*^−/−^, female 4 CTR, 4 *Tph2*^−/−^), and P56 (n = 12: 3 per sex and genotype).

**Figure 1:**
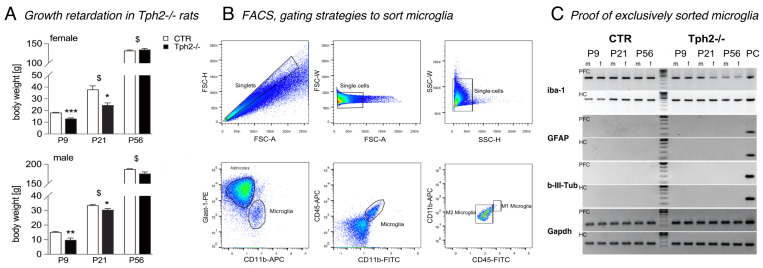
Growth retardation and microglia cell sorting. (**A**) The absence of brain serotonin results in growth retardation in both male and female *Tph2*^−/−^ rats at P9 and P21 and was obsolete at P56; * *p* < 0.05, ** *p* < 0.01, *** *p* < 0.001 indicate statistical significance relative to control littermates (CTR) of the same age, and ^$^ *p* < 0.05 is relative to the previous age. P9 *n* = male 4 CTR, 3 *Tph*^−/−^, female 4 CTR, 4 *Tph2*^−/−^; P21 *n* = male 4 CTR, 3 *Tph2*^−/−^, female 4 CTR, 4 *Tph2*^−/−^; P56 *n* = 3 per sex and genotype. (**B**) Gating strategy plots. To analyze cell phenotypes, a control gate was established using unlabeled cells. Doublets were excluded by gating on FSC-A vs. FSC-H, FSC-H vs. FSC-W, and SSC-H vs. SSC-W parameters. Cells were then gated on FSC-A vs. SSC-A to define the population for further analysis. CD11b vs. Glast1 expression was used to distinguish microglia from astrocytes. Within the CD11b^+^; population, CD45 expression was assessed to determine microglial polarization: CD11b^+^CD45^+^ cells were classified as M1 microglia, while CD11b^+^CD45^−^ cells were considered M2. FSC, forward scatter; SSC, side scatter. (**C**) Verification of purity of sorted microglia by PCR. *Gfap* (170 bp) and βIII-tubulin (*TUBB3,* 129 bp) were used to identify astrocytes and neurons, respectively, while *Iba1* (404 bp) confirmed the presence of microglia. *Gapdh* served as a housekeeping gene. mRNA from whole rat brain homogenate was used as a positive control (PC).

*A priori*, as was previously shown for P2- to P14-old *Tph2*^−/−^ rats [17], lack of brain serotonin leads to transient growth retardation. In our study, the body weights of male and female *Tph2*^−/−^ rats were significantly lower at P9 compared to CTR of the same age (Student’s *t*-test: male 15.3 ± 0.4 vs. 9.9 ± 1.1 g, *p* = 0.0074; female 18.3 ± 0.2 vs. 13.2 ± 0.6 g, *p* = 0.0003; Figure 1A) and at P21 (Student’s *t*-test: 33.6 ± 0.5 g vs. 30.5 ± 0.7 g, *p* = 0.0219; female 38.0 ± 2.7 vs. 24.6 ± 1.7 g, *p* = 0.0266; Figure 1A). By adulthood at P56, no significant difference in body weight was observed (Student’s *t*-test: male 187.8 ± 1.0 vs. 180.0 ± 3.5 g, *p* = 0.0562; female 132.6 ± 1.2 vs. 135.3 ± 2.1 g, *p* = 0.4161; Figure 1A).

### 2.2. Fluorescence-Activated Cell Sorting of Microglia

Upon brain removal, the dissected hippocampus and prefrontal cortex, respectively, were placed into 1.5 mL tubes containing 1 mL of ice-cold, Ca^2+^/Mg^2+^-free HBSS and minced into 1–3 mm pieces for molecular analysis. Tissue was transferred to a 15 mL centrifuge tube with 5 mL of Gibco TrypLE Express (Thermo Fisher Scientific, Dreieich, Germany) and incubated at 37 °C for 5 min. An equal volume of MEM with 10% FBS was added and gently triturated (8–10 times) using a fire-polished glass pipette, and the homogeneous cell suspension was filtered through a 70 μm strainer and centrifuged at 300× *g* for 5 min. After discarding the supernatant, cells were resuspended in cold HBSS containing 1% BSA. An antibody cocktail—CD11b-APC (MA5-17507, Invitrogen, Thermo Fisher Scientific, Dreieich, Germany), CD45-FITC (11-0461-82, Invitrogen), and Glast1-PE (130-118-344, Miltenyi Biotec, Inc., Auburn, CA, USA)—was added at a final 1:500 dilution. Samples were incubated at 4 °C in the dark for 15 min and then centrifuged and washed again with cold HBSS + 1% BSA. To analyze and enrich microglia (Figure 1B), a flow cytometry analyzer (Fortessa (BD), Becton, Dickinson and Company, Franklin Lakes, NJ, USA) and digital cell sorter Aria III (BD) were utilized (core facility, MDC Berlin, Berlin, Germany).

### 2.3. mRNA Expression Analyses

The purity of sorted microglia was confirmed by PCR using cell type-specific markers for astrocytes (*Gfap*), neurons (*Tubb3*), and microglia (Iba1; Table 1), with Gapdh-specific primers used as an internal control. Gel electrophoresis of PCR products showed exclusive amplification of microglial markers and no astrocytic or neuronal markers, confirming the absence of contamination (whole-brain mRNA served as a positive control; Figure 1C). Following verification, RNA was extracted from the sorted microglia using TRIzol (Invitrogen, Carlsbad, CA, USA) according to the manufacturer’s instructions and reverse-transcribed with M-MLV Reverse Transcriptase (Promega, M1701, Madison, WI, USA). mRNA levels of 5-HTRs, BDNF, and cytokines were quantified by real-time PCR using 5X qPCRmix-HS (Evrogen, PK145, Moscow, Russia) on a Bio-Rad CFX96 system. Primer sequences (BIOTEZ-Berlin, Berlin, Germany) are listed in Table 1. Expression levels were normalized to *Gapdh* and are presented as 2^ΔΔCt^ values.

### 2.4. Statistical Analysis

Statistical differences between group means were evaluated by one-way ANOVA, followed by Tukey’s *post hoc* tests in cases where a significant F statistic was obtained (GraphPad PRISM 9 software). For individual comparisons, a Student’s *t*-test was used. All values are expressed as mean ± SEM. *p* values of <0.05 were considered statistically significant.

## 3. Results

### 3.1. Increased Proportion of Microglia in Tph2^−/−^ Rats at P9

Microglia from the hippocampi and prefrontal cortices of male and female CTR and *Tph2*^−/−^ rats at P9, P21, and P56 were sorted based on their CD11b marker expression (Figure 1B). Initially, the fluorescence-detection threshold was set to distinguish CD11b-positive (CD11b^+^) microglia from Glast1^+^ astrocytes. As expected, flow cytometric analysis revealed a high percentage of astrocytes and a low percentage of microglial cells within single-cell suspensions. For example, out of 4,432,756 sorted cells from the hippocampus of P9 male rats, 50.80% were astrocytes, and 1.50% were microglia (Table 2). Notably, the proportion of microglia was higher at P9 compared to later developmental stages. A genotype effect was also observed at this time point, with a significantly higher percentage of CD11b^+^ microglia cells in the hippocampi of both male and female *Tph2*^−/−^ rats compared to WT controls (Student’s *t*-test: male 1.50 ± 0.07 vs. 1.82 ± 0.04%, *p* = 0.0140; female 1.14 ± 0.03 vs. 1.72 ± 0.08, *p* = 0.002; Table 2). Interestingly, at P21, the proportion of astrocytes was significantly higher in the hippocampus of male *Tph2*^−/−^ rats but not in females (Table 2). When microglia were further analyzed based on CD45-FITC and CD11b-APC expression, no effect of the *Tph2*^−/−^ genotype was detected on microglial polarization. The majority of cells in all groups displayed an M0/M2-activated microglial phenotype characterized by CD11b^+^CD45^−^ (Figure 1B).

### 3.2. Distinct Microglial Expression of 5-HT1b, 5-HT2b, 5-HT5a, and 5-HT5b

We extracted mRNA from sorted microglia to determine which of the 5-HTR subtypes are expressed in microglia in the hippocampus and prefrontal cortex of CTR and *Tph2*^−/−^ rats. Among the examined receptors, 5-HT1a, b, d, f; 5-HT2a, b, c; 5-HT4; 5-HT5a, b; 5-HT6; and 5-HT7, we identified four—5-HT1b, 5-HT5a, 5-HT5b, and 5-HT2b—that are expressed in microglia, exhibiting distinct age-related and genotype-dependent expression patterns (Table 3).

### 3.3. Downregulation of Microglial 5-HT1b and 5-HT5b with Age

**Figure 2:**
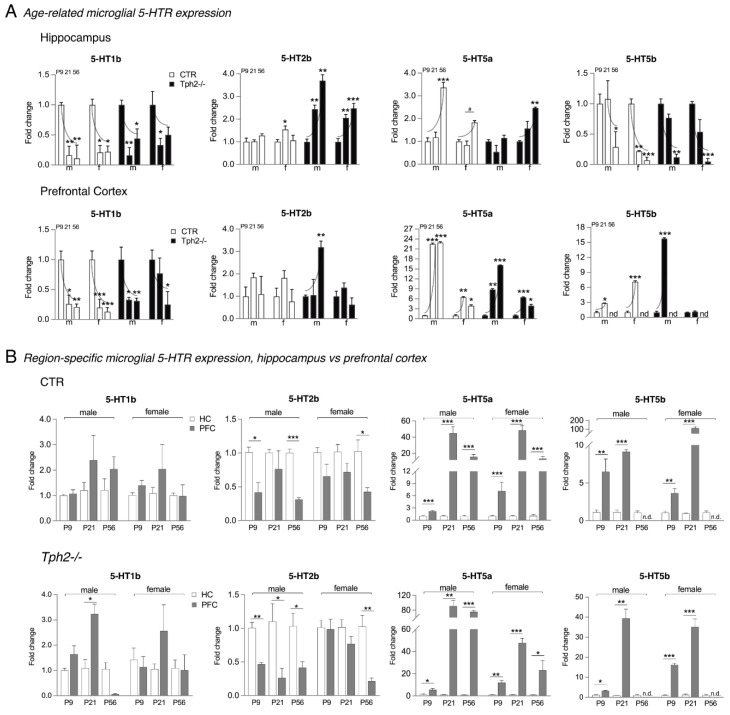
Quantitative analyses of age- and region-specific 5-HTR expression in the hippocampus and prefrontal cortex. (**A**) While 5-HT1b, 5-HT5a, and 5-HT5b mRNA levels vary across age, 5-HT2b is consistently expressed in all groups and significantly upregulated in male and female *Tph2*^−/−^ rats at P21 and P56 compared to control littermates (CTR). An age-dependent decline is observed in 5-HT1b and 5-HT5b expression for all groups, while 5-HT5a is upregulated. In the prefrontal cortex, 5-HT5b expression peaks at P21 and is absent at P56. * *p* < 0.05, ** *p* < 0.01, *** *p* < 0.001 indicate statistical significance relatively to P9; ^#^ *p* < 0.05 Student’s *t*-test, P21 vs. P56. m, male, f, female; P9 *n* = male 4 CTR, 3 *Tph2*^−/−^, female 4 CTR, 4 *Tph2*^−/−^; P21 *n* = male 4 CTR, 3 *Tph2*^−/−^, female 4 CTR, 4 *Tph2*^−/−^; P56 *n* = 3 per sex and genotype. (**B**) 5-HT2b expression peaks in the hippocampus, while 5-HT5a and 5-HT5b levels surge in the prefrontal cortex. 5-HT5b is absent in the prefrontal cortex of male and female CTR and *Tph2*^−/−^ rats at P56. * *p* < 0.05, ** *p* < 0.01, *** *p* < 0.001 significance between regions for the same age.

Quantitative analyses of microglial mRNA levels showed a gradual decline of 5-HT1b and 5-HT5b gene expression in CTR and *Tph2*^−/−^ rats (Figure 2A). Specifically, 5-HT1b, although present at P9, was downregulated at P21 and P56 in both hippocampus (one-way ANOVA: CTR male F(2,8) = 110.0, *p* < 0.0001, CTR female F(2,8) = 45.82, *p* < 0.0001; *Tph2*^−/−^ male F(2,6) = 24.59, *p* = 0.0013; *Tph2*^−/−^ female F(2,8) = 5.643, *p* = 0.0296; Figure 2A) and prefrontal cortex (one-way ANOVA: CTR male F(2,8) = 10.51, *p* = 0.0058, CTR female F(2,8) = 12.29, *p* = 0.0036; *Tph2*^−/−^ male F(2,6) = 17.61, *p* = 0.0031, *Tph2*^−/−^ female (2,8) = 1.430, *p* = 0.2944, Student’s *t*-test P9 vs. P56, *p* = 0.045; Figure 2A). Gene expression of 5-HT5b remained similar in male hippocampi at ages P9 and P21, but there was a significant downregulation in male and female rats at P56 (one-way ANOVA: CTR male F(2,8) = 2.570, *p* = 0.1374, Student’s *t*-test: P21 vs. P56, *p* = 0.0208, CTR female F(2,8) = 18.63, *p =* 0.0010; *Tph2*^−/−^ male F(2,6) = 6.989, *p* = 0.0271; *Tph2*^−/−^ female F(2,8) = 7.785, *p* = 0.0133; Figure 2A). In the prefrontal cortex, levels of 5-HT5b peaked at P21, showing fold changes ranging from 3 to 15 times higher compared to P9 (Student’s *t*-test: CTR male 1.00 ± 0.22 vs. 2.86 ± 0.04, *p* = 0.0440, CTR female 1.00 ± 0.28 vs. 7.15 ± 0.20, *p* = 0.0002; *Tph2*^−/−^ male 1.00 ± 0.26 vs. 15.78 ± 0.17, *p* = 0.0077; Figure 2A). In female *Tph2*^−/−^ rats, no increase at P21 was observed (1.00 ± 0.05 vs. 1.17 ± 0.13, *p* = 0.2823; Figure 2A). Notably, 5-HT5b was absent in the adult rats prefrontal cortex at P56 regardless of the genotype (Figure 2A).

### 3.4. Upregulation of Microglial 5-HT2b and 5-HT5a with Age

The observed downregulation of 5-HT5b expression in the hippocampus of CTR and *Tph2*^−/−^ rats’ microglia parallels an upregulation of 5-HT5a. Levels of 5-HT5a were significantly increased at P56 (one-way ANOVA: CTR male F(2,8) = 20.59, *p* = 0.0007, CTR female F(2,8) = 3.350, *p* = 0.0877, Student’s *t*-test: P21 vs. P56, *p* = 0.0291; *Tph2*^−/−^ female F(2,8) = 7.46, *p* = 0.0148; Figure 2A). In male *Tph2*^−/−^ rats, 5-HT5a gene expression was similar in all groups (F(2,8) = 1.632, *p* = 0.2718; Figure 2A). In the prefrontal cortex, microglial 5-HT5a levels were strongly upregulated at P21 and P56 (one-way ANOVA: CTR male F(2,8) = 15.92, *p* = 0.0016, CTR female F(2,8) = 17.14, *p* = 0.0013; *Tph2*^−/−^ male F(2,8) = 36.03, *p* = 0.0011, *Tph2*^−/−^ female F(2,8) = 9.612, *p* = 0.0075; Figure 2A). Specifically, fold changes were >20 times higher in male CTR rats (Tukey’s *post hoc* test: P9 1.00 ± 0.07 vs. P21 22.51 ± 0.24, *p* < 0.0001, vs. P56 22.93 ± 0.20, *p* < 0.0001; Figure 2A) and 9 to 15 times higher in male *Tph2*^−/−^ rats (Tukey’s *post hoc* test: P9 1.00 ± 0.23 vs. P21 8.83 ± 0.25, *p* = 0.0043, vs. P56 16.11 ± 0.08, *p* = 0.0006; Figure 2A). Expression in both female CTR and *Tph2*^−/−^ rats revealed the highest expression levels with a fold change of more than six at P21 (Tukey *post hoc* tests: CTR *p* = 0.0021, *Tph2^−/−^ p* = 0.0001; Figure 2A).

Gene expression levels of 5-HT2b in the hippocampus and prefrontal cortex of CTR rats remained consistent across groups (Figure 2A). However, in the lack of brain serotonin, 5-HT2b levels gradually rose in the hippocampus during both youth and adulthood (one-way ANOVA: *Tph2*^−/−^ male F(2,6) = 10.08, *p* = 0.0121, female (F(2,8) = 17.41, *p* = 0.0012; Figure 2A). Specifically, 5-HT2b mRNA was upregulated two to four times in male *Tph2*^−/−^ (Tukey’s *post hoc* test: P9 1.00 ± 0.12 vs. P21 2.44 ± 0.18, *p* = 0.0042, vs. P56 3.70 ± 0.26, *p* = 0.0028; Figure 2A) and female rats (Tukey’s *post hoc* test: P9 1.00 ± 0.14 vs. P21 2.06 ± 0.14, *p* = 0.0021, vs. P56 2.48 ± 0.22, *p* = 0.0008; Figure 2A) compared to P9 of the same sex. In the prefrontal cortex, a significant three-fold upregulation was observed at P56 in male *Tph2*^−/−^ rats (one-way ANOVA: F(2,6) = 4.445, *p* = 0.0654, Student’s *t*-test: P9 vs. P56, *p* = 0.0042; Figure 2A).

### 3.5. 5-HT2b Expression Peaks in the Hippocampus

When we looked at the region-specific expression distribution, i.e., hippocampus vs. prefrontal cortex, we observed no significant differences in the levels of 5-HT1b in CTR rats. Nevertheless, there was a tendency towards higher 5-HT1b expression in the male and female prefrontal cortex at P21 (Figure 2B). Similar results were found for *Tph2*^−/−^ rats, with significantly higher expression levels detected in the male prefrontal cortex at P21 (Student’s *t*-test *p* = 0.0143; Figure 2B); expression levels were down at P56. In contrast, 5-HT2b expression peaked in the hippocampus of male and female CTR and *Tph2*^−/−^ rats. Specifically, 5-HT2b levels in male hippocampi were two to three times higher compared to the prefrontal cortex (Student’s *t*-test: CTR P9 *p* = 0.1009, P56 *p* = 0.0004; *Tph2*^−/−^ P9 *p* = 0.0033, P21 *p* = 0.0480, P56 *p* = 0.0421; Figure 2B). In female rats at P56, gene expression in the prefrontal cortex was two to four times lower compared to the hippocampus (Student’s *t*-test: P56 CTR *p* = 0.0280; *Tph2^−/−^ p* = 0.0095; Figure 2B).

### 3.6. 5-HT5a Levels Surge in the Prefrontal Cortex

The expression of 5-HT5a and 5-HT5b was significantly higher in the prefrontal cortex compared to the hippocampus. Specifically, 5-HT5a was upregulated by two to 40 times across age groups in the prefrontal cortex of male and female CTR rats (Student’s *t*-test: male P9 *p* = 0.0005, P21 *p* = 0.0017, P56 *p* = 0.0062; female P9 *p* = 0.0314, P21 *p* = 0.0002, P56 *p* = 0.0130; Figure 2B) and even seven to 80 times in *Tph2*^−/−^ rats compared to the hippocampus (Student’s *t*-test: male P9 *p* = 0.0358, P21 *p* = 0.0044, P56 *p* ≤ 0.0001; female P9 *p* = 0.0019, P21 *p* ≤ 0.0001, P56 *p* = 0.0130; Figure 2B). Likewise, 5-HT5b gene expression was upregulated four to 40 times in the prefrontal cortex of male and female CTR and *Tph2*^−/−^ rats (Student’s *t*-test: CTR male P9 *p* = 0.0131, P21 *p* < 0.0001, female P9 *p* = 0.0071, P21 *p* = 0.0002; *Tph2*^−/−^ male P9 *p* = 0.0153, P21 *p* = 0.0012, female P9 *p* < 0.0001, P21 *p* = 0.0002; Figure 2B). In female CTR rats, upregulation was even 100 times the expression levels in the hippocampus. Notably, 5-HT5b was absent in the prefrontal cortex of male and female CTR and *Tph2*^−/−^ rats at P56 (Figure 2B).

### 3.7. A Strong Upregulation of 5-HT2b Gene Expression and BDNF in Tph2^−/−^ Rats

**Figure 3:**
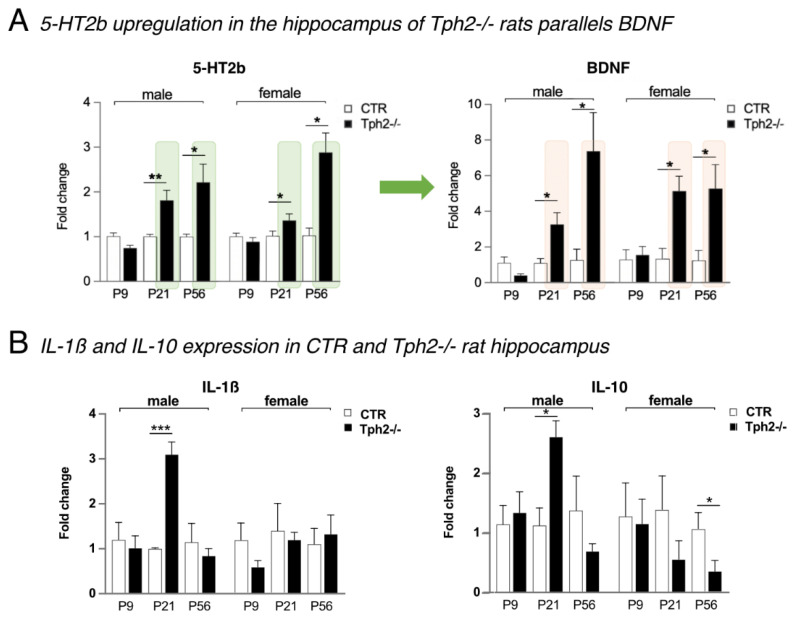
Microglial 5-HT2b, BDNF, and cytokine signaling in the hippocampus. (**A**) Strong upregulation of 5-HT2b in the hippocampus of *Tph2*^−/−^ rats was accompanied by a significant increase observed in microglial BDNF expression at P21 and P56 compared to control littermates (CTR). * *p* < 0.05, ** *p* < 0.01 indicate statistical significance between genotypes. P9 *n* = male 4 CTR, 3 *Tph2*^−/−^, female 4 CTR, 4 *Tph2*^−/−^; P21 *n* = male 4 CTR, 3 *Tph2*^−/−^, female 4 CTR, 4 *Tph2*^−/−^; P56 *n* = 3 per sex and genotype. (**B**) Lack of brain serotonin significantly upregulates IL-1b and IL-10 in the hippocampus of male *Tph2*^−/−^ rats at P21, while IL-10 is downregulated in females at P56. * *p* < 0.05, *** *p* < 0.001 indicate statistical significance between genotypes of the same age.

Earlier studies have shown that in the absence of brain serotonin, BDNF protein levels are upregulated in the adult hippocampus and prefrontal cortex [18,19,20]. The hippocampus, in particular, is hyper-innervated by serotonin fibers [18]. In the experiments here, we detected strong 5-HT2b gene expression in the hippocampus in CTR and a significant upregulation of 5-HT2b in *Tph2*^−/−^ rats (Figure 3A). At P21, both male and female *Tph2*^−/−^ rats had 1.5 to two times more 5-HT2b compared to CTR (Student’s *t*-test: male *p* = 0.0050, female *p* = 0.050; Figure 3A). The difference grew with age: at P56, levels were two to three times higher (Student’s *t*-test: male *p* = 0.0158, female *p* = 0.0086; Figure 3A). Excitingly, microglial BDNF in the hippocampus was also significantly increased and tripled in *Tph2*^−/−^ male rats at P21 (Student’s *t*-test *p* = 0.0235) and doubled again at P56 compared to male CTR (*p* = 0.0345; Figure 3A). In female rats, BDNF expression was increased five times at both P21 (*p* = 0.0292) and P56 (*p* = 0.0470; Figure 3A) compared to CTR.

### 3.8. Expression Profiles of IL-1β and IL-10 in the Absence of Brain Serotonin

Based on the above results for the hippocampus, we measured how the expression levels of two cytokines, IL-1β (pro-inflammatory) and IL-10 (anti-inflammatory), behaved in the absence of brain serotonin. In male *Tph2*^−/−^ rats at P21, both IL-1β (Student’s *t*-test: 1.0 ± 0.02 vs. 3.1 ± 0.28, *p* = 0.0003; Figure 3B) and IL-10 (Student’s *t*-test: 1.12 ± 0.4 vs. 2.60 ± 0.27, *p* = 0.0162; Figure 3B) were significantly upregulated in microglia of the hippocampus. No further difference was observed for IL-1β. IL-10, on the other hand, was downregulated in female *Tph2*^−/−^ rats at P21 (Student’s *t*-test: 1.40 ± 0.56 vs. 0.57 ± 0.30, *p* = 0.1133; Figure 3B) and significantly at P56 (Student’s *t*-test: 1.08 ± 0.14 vs. 0.37 ± 0.22, *p* = 0.0453; Figure 3B), which is in contrast to the upregulation of anti-inflammatory BDNF.

## 4. Discussion

We report the selective and transient gene expression of 5-HTR subtypes in microglia, revealing a novel layer of serotonin-mediated regulation beyond its traditional context. We identified four microglial 5-HTR subtypes expressed in the hippocampus and prefrontal cortex, while mRNA levels for other subtypes were undetectable. Specifically, 5-HT5a and 5-HT2b were upregulated during postnatal development, whereas 5-HT1b and 5-HT5b were transiently expressed early and subsequently downregulated. Notably, in the lack of brain serotonin, we found that the proportion of microglial cells was significantly higher in both male and female rats at P9, suggesting a compensatory mechanism to serotonin deficiency during early neurodevelopment. At later stages, microglial 5-HT2b expression was strongly upregulated in *Tph2*^−/−^ rats, particularly in the hippocampus, mirroring the expression pattern of BDNF. These dynamic shifts in expression might reflect changes in microglial signaling capacity, enabling temporal and stage-specific interactions with their environment, and underscore the complexity of serotonin-mediated regulation.

Traditionally, 5-HTRs have been thought to be primarily neuronal, mediating a wide range of neuromodulator functions. However, recent data—including our own—demonstrate that microglia exhibit distinct, cell type-specific expression patterns of 5-HTR subtypes, which are modulated by environmental stressors. For instance, neurons in the mouse dentate gyrus predominantly express 5-HT1a, 5-HT2a, 5-HT2c, and 5-HT5a [10,11,12], whereas cultured microglia express 5-HT2b and 5-HT5a [15,21,22]. Targeting 5-HT1a with an antagonist affected receptor activity in the hippocampus and cortex [23]; the receptor is also downregulated under stressful behavioral conditions [24,25]. In contrast, 5-HT5b expression observed on CA1 pyramidal neurons was upregulated in young adult male rats following stress or testosterone injection [24,26]. These patterns highlight the dynamic and stress-sensitive nature of serotonin signaling across cell types.

5-HT2b is critical for microglial serotonin sensing during early postnatal development, and its absence primes microglia toward a more reactive, pro-inflammatory state [22,27]. Furthermore, aberrant 5-HT2b signaling in pathological conditions such as cerebral ischemia-reperfusion injury has been shown to promote M1 polarization and neuroinflammation [28]. At P21, we observed elevated IL-1β and IL-10 expression in *Tph2*^−/−^ male rats’ hippocampus and reduced IL-10 levels in females, indicating a shift toward pro-inflammatory conditions. Serotonin has been shown to suppress IL-1β expression in human macrophages [29], and its deficiency may directly contribute to this upregulation. Previous studies revealed that serotonin, particularly via 5-HT2b, inhibits LPS-induced pro-inflammatory cytokine release [21] without affecting IL-10 levels [29], suggesting that IL-10 upregulation involves other mechanisms. BDNF often promotes IL-10 release [8], as demonstrated in multiple sclerosis studies [30], indicating a protective feedback mechanism. However, our data show no change (males) and reduced IL-10 levels (females) in the absence of brain serotonin, while BDNF is upregulated, possibly reflecting an attempt to restore inflammatory balance. This may contribute to the increased number of microglia observed at P9 [31]. The upregulation of BDNF may result from adaptive mechanisms triggered by serotonin deficiency, as our previous findings showed elevated BDNF levels in the hippocampus and prefrontal cortex in *Tph2*^−/−^ mice [19] and rats likely due to microglial release. Thus, the balance between BDNF and cytokine signaling is crucial for regulating microglial activation and inflammation.

Our findings reveal distinct temporal expression of microglial serotonin and BDNF, which might affect microglial function [32]. Acute serotonin exposure, such as following LPS, attenuates pro-inflammatory pathways without affecting IL-10 [33], while chronic serotonin signaling might be essential for maintaining IL-10 production, as shown here for female rats. We hypothesize that chronic serotonin depletion might impair microglia’s full anti-inflammatory capacity—which is shifting to an anti-inflammatory phenotype, i.e., expressing IL-10 following the removal of toxic molecules [34]. While serotonin is specifically required for optimal IL-10 production, BDNF promotes the morphological CD11b^+^CD45^−^ (M0/M2) characteristic. Additionally, serotonin deficiency may disrupt BDNF’s ability to stimulate IL-10 release, suggesting serotonin acts as a co-factor in this process. Notably, in chronic inflammation, such as Alzheimer’s disease, both 5-HT2b and BDNF levels are elevated, paradoxically contributing to neurotoxicity as BDNF released near amyloid plaques promotes TNF-α and glutamate release [35,36,37].

Sex differences in the expression of receptors or pro-inflammatory factors may be due to stronger innate immune activation in males, while females often show downregulation of anti-inflammatory mediators, possibly influenced by hormonal modulation of microglial activity. Despite differing mechanisms, both patterns reflect a shift toward a pro-inflammatory state.

## 5. Conclusions

Overall, our findings suggest that lack of brain serotonin drives an age- and sex-dependent inflammatory state, with upregulation of 5-HT2b and BDNF in both male and female rats acting as compensatory mechanisms. However, altered IL-1b and IL-10 expression, particularly the reduction in females, indicates that the functional benefits of this response remain unclear.

## Figures and Tables

**Table 1:** Real-time PCR primer sequences.

Gene	Forward (5′-3′)	Reverse (5′-3′)
*5-ht1a*	CACTTGGCTCATTGGCTTTC	CGAAAGTGGAGTAGATGGTGTAG
*5-ht1b*	CGTCCTCTACACGGTCTACT	CGGGCTTCCACATAGATAC
*5-ht1d*	GCCTTCTACATCCCATCCATC	CGCAGAGCCCGTGATAAG
*5-ht1f*	CACCACCCAGCCAACTATTTA	CACAGAGTCCTTGTCCCATAATC
*5-ht2a*	TCCAGAGATGCTAACACTTCG	CCTGGAGATGAAGAATGGAGAG
*5-ht2b*	GAATAGAGGCTGATGTGGTCAA	CAAAGCGTGAATGGTGAGAAAG
*5-ht3a*	GAACACCAGAAGAAGTGAGGTC	GAAGATACTGGGCAGCAAGAG
*5-ht3b*	CGAGAGGTTTGGAATGATGAGT	GGATGGGCTTGTGGTTTCTA
*5-ht4*	CTGAGACTAGGAGGTGGTAGG	GCTTTCCTACTGACCTGAACC
*5-ht5a*	AGGGAACAGAAGGAGCAAAG	GTAGATGAGCGGGTTGAAGAA
*5-ht5b*	GAACTACAACAATGCCTTCAAGAG	CGGTGTGATTTCTGGAGTGT
*5-ht6*	CTGAGACTAGGAGGTGGTAGG	GCTTTCCTACTGACCTGAACC
*5-ht7*	CCATCACCTTACCTCCTCTCT	ACACTCTTCCACCTCCTTCT
*Bdnf*	GAGACAAGAACACAGGAGGAAA	CCCAAGAGGTAAAGTGTAGAAGG
*Il-10*	CTGCTATGTTGCCTGCTCTTA	GGAACTGAGGTATCAGAGGTAATAAATA
*Il-1β*	GCAATGGTCGGGACATAGTT	GTAAGTGGTTGCCTGTCAGAG
*Gapdh*	GCTGTGGGCAAGGTCATCC	CTTCACCACCTTCTTGATGTC
*Gfap*	GTGTGGAGTGCCTTCGTATTAG	GAAGGTTAGCAGAGGTGACAAG
*Tubb3*	GGAACGCATCAGTGTCTACTAC	GGCCTGAATAGGTGTCCAAA
*Iba1*	ATGAGCCAGAGCAAGGATTT	GTTGGCTTCTGGTGTTCTTTG

**Table 2:** Microglia vs. astrocytes distribution.

**Male %**	**Hippocampus**		**Female %**	**Hippocampus**	
CTR	Mic	Astro	CTR	Mic	Astro
P9	1.50 (0.07)	50.80 (3.13)	P9	1.14 (0.03)	59.18 (1.75)
P21	0.72 (0.12)	56.65 (1.27)	P21	0.50 (0.10)	55.25 (3.86)
P56	0.57 (0.13)	45.67 (0.57)	P56	0.61 (0.24)	45.13 (1.08)
*Tph2* ^−/−^			*Tph2* ^−/−^		
P9	1.82 (0.04) *	48.43 (4.22)	P9	1.72 (0.08) ***	50.95 (4.59)
P21	0.50 (0.02)	74.83 (1.37) ***	P21	0.75 (0.08)	52.05 (0.23)
P56	0.37 (0.11)	45.03 (0.23)	P56	1.66 (0.75)	44.10 (0.23)
					
**Male %**	**Prefrontal Cortex**		**Female %**	**Prefrontal Cortex**	
CTR	Mic	Astro	CTR	Mic	Astro
P9	1.56 (0.08)	59.28 (7.86)	P9	1.36 (0.10)	46.33 (3.52)
P21	0.66 (0.08)	55.43 (2.38)	P21	0.75 (0.07)	52.33 (0.54)
P56	0.63 (0.07)	43.87 (0.84)	P56	0.48 (0.11)	45.10 (1.05)
*Tph2* ^−/−^			*Tph2* ^−/−^		
P9	1.70 (0.04)	63.63 (4.19)	P9	1.40 (0.03)	45.45 (6.48)
P21	0.61 (0.05)	68.83 (5.13) ^#^	P21	0.71 (0.05)	51.43 (0.21)
P56	0.73 (0.27)	45.40 (0.35)	P56	0.50 (0.03)	43.93 (1.85)

Percentage of all cells (SEM); * *p* < 0.05, *** *p* < 0.001 to CTR of same age, cell type, and brain region, ^#^ *p* = 0.0556.

**Table 3:** Quantitative expression of 5-HTR subtypes in microglia in the hippocampus (HC) and prefrontal cortex (PFC).

**HC**	1a	**5-HT1b**		1d	1f	2a	**5-HT2b**		4	**5-HT5a**		**5-HT5b**		6	7
male		*CTR*	*Tph2* ^−/−^				*CTR*	*Tph2* ^−/−^		CTR	*Tph2* ^−/−^	CTR	*Tph2* ^−/−^		
P9	n.d.	∗∗∗	∗∗∗	n.d.	n.d.	n.d.	∗	∗	n.d.	∗	∗	∗∗∗	∗∗∗	n.d.	n.d.
P21	n.d.	∗	∗	n.d.	n.d.	n.d.	∗	∗∗∗	n.d.	∗	∗	∗∗∗	∗∗∗	n.d.	n.d.
P56	n.d.	∗	∗	n.d.	n.d.	n.d.	∗	∗∗∗	n.d.	∗∗∗	∗∗∗	∗	∗	n.d.	n.d.
female															
P9	n.d.	∗∗∗	∗∗∗	n.d.	n.d.	n.d.	∗	∗	n.d.	∗	∗	∗∗∗	∗∗∗	n.d.	n.d.
P21	n.d.	∗	∗	n.d.	n.d.	n.d.	∗∗	∗∗∗	n.d.	∗	∗∗	∗	∗∗	n.d.	n.d.
P56	n.d.	∗	∗	n.d.	n.d.	n.d.	∗	∗∗∗	n.d.	∗∗∗	∗∗∗	∗	∗	n.d.	n.d.
															
**PFC**	1a	**5-HT1b**		1d	1f	2a	**5-HT2b**		4	**5-HT5a**		**5-HT5b**		6	7
male		CTR	*Tph2* ^−/−^				CTR	*Tph2* ^−/−^		CTR	*Tph2* ^−/−^	CTR	*Tph2* ^−/−^		
P9	n.d.	∗∗∗	∗∗∗	n.d.	n.d.	n.d.	∗	∗	n.d.	∗	∗	∗	∗	n.d.	n.d.
P21	n.d.	∗	∗	n.d.	n.d.	n.d.	∗∗	∗	n.d.	∗∗∗∗	∗∗	∗∗	∗∗∗	n.d.	n.d.
P56	n.d.	∗	∗	n.d.	n.d.	n.d.	∗	∗∗∗	n.d.	∗∗∗∗	∗∗∗∗	n.d.	n.d.	n.d.	n.d.
female															
P9	n.d.	∗∗∗	∗∗∗	n.d.	n.d.	n.d.	∗	∗	n.d.	∗	∗	∗	∗	n.d.	n.d.
P21	n.d.	∗	∗∗	n.d.	n.d.	n.d.	∗∗	∗	n.d.	∗∗∗	∗∗∗	∗∗∗	∗	n.d.	n.d.
P56	n.d.	∗	∗	n.d.	n.d.	n.d.	∗	∗	n.d.	∗∗	∗∗	n.d.	n.d.	n.d.	n.d.

* low, **, ***, and **** high indicate relative amounts compared to each other, n.d. = not detected.

## Data Availability

The original contributions presented in this study are included in the article. Further inquiries can be directed to the corresponding author(s).
